# Transcriptional Changes Associated with Long-Term Left Ventricle Volume Overload in Rats: Impact on Enzymes Related to Myocardial Energy Metabolism

**DOI:** 10.1155/2015/949624

**Published:** 2015-10-25

**Authors:** Elise Roussel, Marie-Claude Drolet, Elisabeth Walsh-Wilkinson, Wahiba Dhahri, Dominic Lachance, Suzanne Gascon, Otman Sarrhini, Jacques A. Rousseau, Roger Lecomte, Jacques Couet, Marie Arsenault

**Affiliations:** ^1^Groupe de Recherche sur les Valvulopathies, Centre de Recherche, Institut Universitaire de Cardiologie et de Pneumologie de Québec, Université Laval, Quebec City, QC, Canada G1V 4G5; ^2^Sherbrooke Molecular Imaging Center, Research Center of Centre Hospitalier Universitaire de Sherbrooke (CRCHUS), Université de Sherbrooke, Sherbrooke, QC, Canada J1H 5N4

## Abstract

Patients with left ventricle (LV) volume overload (VO) remain in a compensated state for many years although severe dilation is present. The myocardial capacity to fulfill its energetic demand may delay decompensation. We performed a gene expression profile, a model of chronic VO in rat LV with severe aortic valve regurgitation (AR) for 9 months, and focused on the study of genes associated with myocardial energetics. *Methods*. LV gene expression profile was performed in rats after 9 months of AR and compared to sham-operated controls. LV glucose and fatty acid (FA) uptake was also evaluated *in vivo* by positron emission tomography in 8-week AR rats treated or not with fenofibrate, an activator of FA oxidation (FAO). *Results*. Many LV genes associated with mitochondrial function and metabolism were downregulated in AR rats. FA *β*-oxidation capacity was significantly impaired as early as two weeks after AR. Treatment with fenofibrate, a PPAR*α* agonist, normalized both FA and glucose uptake while reducing LV dilation caused by AR. *Conclusion*. Myocardial energy substrate preference is affected early in the evolution of LV-VO cardiomyopathy. Maintaining a relatively normal FA utilization in the myocardium could translate into less glucose uptake and possibly lesser LV remodeling.

## 1. Introduction

Aortic regurgitation (AR) is associated with a long asymptomatic period during which the left ventricle (LV) progressively dilates and hypertrophies in response to chronic volume overload. This process is accompanied by a decrease in LV function, occurrence of symptoms, and eventually heart failure [1]. No medical therapy has yet been clearly shown to be effective to slow dilation, hypertrophy, and loss of function or to have any impact on morbidity or mortality [2]. Chronic AR often secondary to rheumatic fever is a condition still frequent in developing countries and in populations having less than adequate access to health care [3, 4].

Gene expression profiles have been established in several animal models of LV eccentric hypertrophy, including by us in a rat model after two weeks of severe AR, a period characterized with intense LV remodeling [5–8]. A similar profile has not been performed at a later stage of the disease. Considering that AR is a chronic condition often evolving over decades in human, the study of animals later in the disease is of great interest. Contrary to the fast evolution of other VO models such as aortocaval fistula (ACF), severe AR in rats is associated with important LV hypertrophy and dilation, moderate loss of systolic function, diastolic dysfunction, and a low rate of congestive heart failure [9–11]. Significant LV fibrosis and increased myocardial collagen content are present later in the evolution of this disease which is associated with increased mortality [9].

Abnormalities in energy metabolism in the rat AR model are consistent with a pattern of substrate utilization favoring glucose instead of fatty acid oxidation (FAO) [12–14]. These changes have been associated with a general decrease in the activity of enzymes implicated in FAO and PPAR*α* expression, a transcription factor controlling a number of genes implicated in this process [14, 15].

Here, we present LV gene expression profiling late (9 months) during the evolution of this eccentric hypertrophy model caused by severe aortic valve regurgitation in male Wistar rats. We show a general downregulation of genes involved in fatty acid oxidation and bioenergetics. These anomalies occur early in the disease and result in observable changes of* in vivo* myocardial substrate preference as investigated by micropositron emission tomography. We also demonstrate that this can be countered by treating AR rats with a PPAR*α* agonist, fenofibrate.

## 2. Methods

### 2.1. Animal Experiments

Six groups of Wistar male rats (350–375 g) purchased from Charles River (Saint-Constant QC, Canada) were studied for either 2, 14, or 270 days. For each end-point time, the animals were divided in two groups: sham-operated animals or AR. Groups were composed of 8 animals with the exception of the 270-day AR group which is composed of 15 animals. The protocol was approved by the Université Laval's Animal Protection Committee and followed the recommendations of the Canadian Council on Laboratory Animal Care. Severe AR was induced by retrograde puncture of the aortic valve leaflets under echocardiographic guidance as previously described [16–18]. Only animals with >65% regurgitation were included in the study. A complete echo exam was performed before AR induction and at the end of the protocol as previously described [13, 14]. Left ventricular and arterial pressures and *dP*/*dt* (positive and negative) were measured invasively using a dedicated catheter under 1.5% isoflurane anesthesia (5 animals/gr.) [10, 11, 16]. The hearts were harvested as previously described [13].

### 2.2. Microarray Analysis

Total LV RNA was extracted from stored LV tissues (Sham and AR-sed (*n* = 5/group)) as previously described [8]. The biotin-labeled cRNA preparations were hybridized to BeadChip RatRef-12 microarrays (Illumina; San Diego, CA) according to supplier's protocol (11286340 rev. A), using 750 ng per array. After hybridization and washes, arrays were incubated in streptavidin-Cy3 solution and washed, and fluorescence data were collected on a BeadArray reader (Illumina). Treatment of data was performed with the FlexArray software package (version 1.6.3, http://genomequebec.mcgill.ca/FlexArray). Raw fluorescence data were processed and normalized with the lumi Bioconductor package (http://bioconductor.org/) version 1.1.0., and differential expression was determined according to the random variance model of Wright and Simon (SAM analysis) [19]. Complete data (complying with MIAME guidelines) are available at the GEO database (NCBI) under the Accession number GSE17050. Genes were considered regulated when their fold change value was greater than 1.5 or less than 0.67. The change *p* value had to be below 0.01 for regulated genes. The comparative analysis of expression data using the gene ontology (GO) vocabulary was performed using the EASE software [20].

### 2.3. Analysis of mRNA Accumulation by Quantitative RT-PCR

The analysis of LV mRNA levels by quantitative RT-PCR has been described in detail elsewhere [8]. QuantiTect and IDT (Coralville, Iowa) Primer Assays (preoptimized specific primer pairs (see Tables 1 and Supplementary Table S1 in Supplementary Material available online at http://dx.doi.org/10.1155/2015/949624)) and QuantiFast SYBR Green PCR kits (Qiagen) were used. We also used one pair of nonpreoptimized primers for ECHS1 (5′-GCTTTCAGGGTGTCTTGATTTG-3′ and 5′-GAGCTATGCACTGCAGATAGT-3′; 95 bp transcript). Cyclophilin A (PPIA) was used as the control “housekeeping” gene.

### 2.4. Enzyme Activity Determination

Left ventricle samples were kept at −80°C until assayed for maximal (*V*_max_) enzyme activities as described elsewhere [12–15].

### 2.5. Mitochondrial DNA Quantification

LV tissue DNA was isolated using standard procedure and ten nanograms of each sample were analyzed in triplicate using the QuantiFast SYBR Green PCR kit. QuantiTect primers (QT00371308) for the rat Edn1 intronless gene were used for the relative quantification of nuclear DNA, whereas the mitochondrial DNA was quantified with a rat Cox1 (GenBank AY172581) specific primer pair: forward, 5′-AGAAGCTGGAGCTGGAACAG-3′; reverse, 5′-AGATAGAAGACACCCCGGCT-3.

The relative cell mitochondrial DNA copy number was calculated in a similar way as for gene expression analysis.

### 2.6. Staining for Capillaries Density Measurement

Sections of 8 *μ*m thickness were cut from the frozen left ventricle and were stained with isolectin B4 from* Bandeiraea simplicifolia* coupled with horseradish peroxidase (Sigma, Mississauga, ON, Canada), and capillary density was analyzed in the subendocardial region of the LV myocardium (inner third) as described elsewhere [21].

### 2.7. Small Animal *μ*PET Protocol

Adult male Wistar rats were divided into 3 groups as follows: (1) Sham-operated control animals (Sham; *n* = 5); (2) AR controls (AR; *n* = 5); (3) AR rats treated with fenofibrate (100 mg/kg/day PO in unsweetened fruit gelatin daily; SF; *n* = 5). Fenofibrate was started one week before surgery and continued for 9 weeks until sacrifice [15]. Imaging experiments and data analysis were performed essentially as described before [13, 14, 22–26] on a LabPET avalanche photodiode-based small animal PET scanner (Gamma Medica, Northridge, CA) at the Sherbrooke Molecular Imaging Centre. [^18^F]-fluorodeoxyglucose ([^18^F]-FDG) or [^18^F]-fluorothioheptadecanoic acid ([^18^F]-FTHA) (30–40 MBq, in 0.3 mL plus 0.1 mL flush of 0.9% NaCl) was injected via the caudal vein over 30 s. A 45 min dynamic PET data acquisition followed by a 15 min static acquisition was done to determine glucose utilization [myocardial metabolic rate of glucose (MMRG)] using multicompartmental analysis as previously described [25, 27]. The static scan served to draw regions-of-interest (ROIs) on each segment of the LV wall. Blood samples were taken before and after the scans to determine an average blood glucose level. In another experiment, a 45 min dynamic acquisition with [^18^F]-FTHA was used to determine myocardial nonesterified fatty acid (NEFA) uptake (*K*_*m*_). Myocardial NEFA fractional uptake (*K*_*i*_) was determined by a Patlak graphical analysis of the [^18^F]-FTHA data.

### 2.8. Statistical Analysis

Results are presented as mean ± SEM unless specified otherwise. Intergroup comparisons were done using Student's *t*-test or Mann-Whitney *t*-test for *μ*PET protocol. One-way was also used for the analysis of data when required with Dunnett's posttest. Statistical significance was set at a *p* < 0.05. Data and statistical analysis were performed using Graph Pad Prism version 6.04 for Windows, Graph Pad Software (San Diego, CA).

## 3. Results

After 9 months, eight of fifteen (8/15) AR animals were still alive whereas all sham-operated animals were alive. No differences in body weight were observed between the sham and AR groups. Overall growth was similar between groups (similar tibial lengths, results not shown). Indexed wet heart tissue weights were significantly increased in the AR group compared to controls (Table 2).

### 3.1. Hemodynamics

Systolic arterial pressure was similar between 9-month AR and sham-operated rats (Table 2). As expected, diastolic arterial pressure was significantly lower in AR animals resulting in a significantly increased pulse pressure and lowered mean arterial pressure.

Invasive measurements showed a decrease in both negative (an index of diastolic function) and positive (systolic function) *dP*/*dt* in AR after 9 months (35% and 24%, resp.). Left ventricular end-diastolic pressure was significantly increased in AR rats.

### 3.2. Echocardiographic Data

LV end-diastolic and end-systolic diameters were significantly increased in AR rats (Table 3). Stroke volume was also increased. The same was true for diastolic LV wall thickness.

### 3.3. Microarray Study

**Figure 1:**
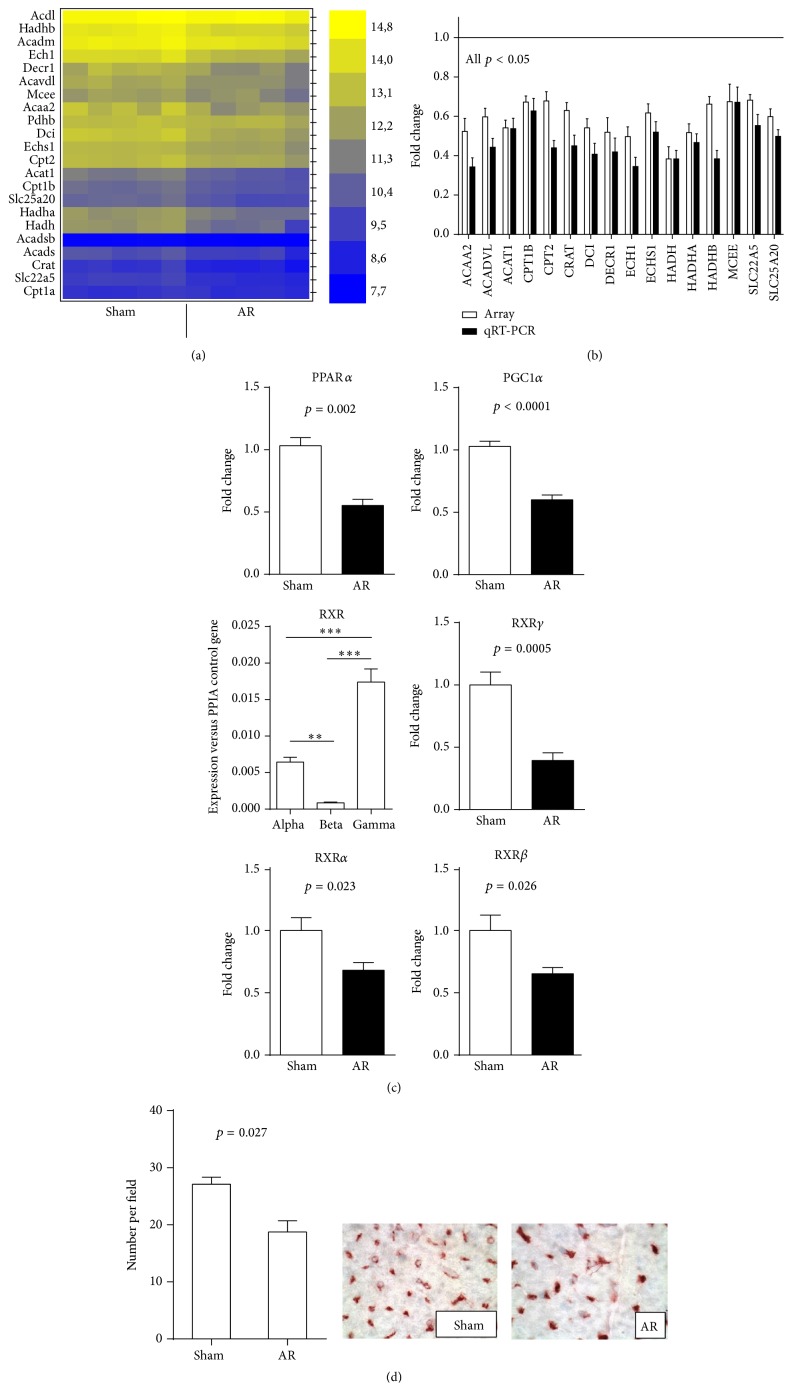
(a) Heat map of expression of 22 genes associated with FAO in LV of 9-month rats. Levels of expression are illustrated from the highest (bright yellow) to the lowest (dark blue). Five animals were studied per group and their expression levels are illustrated individually. (b) Comparison between fold change results obtained from the microarray and by quantitative RT-PCR for a subset of 16 genes illustrated in (a). Results are reported in fold change compared to sham controls as the mean ± SEM (*n* = 5 per group for array and *n* = 6 for qRT-PCR). Levels in sham animals were fixed to 1. (c) PPAR*α*, PGC1*α*, and RXRs LV mRNA levels in 9-month AR rats are strongly downregulated. Exact *p* values are indicated when two groups were compared. ^*∗∗*^ *p* < 0.01 and ^*∗∗∗*^ *p* < 0.001 between groups. (d) Evaluation of capillaries density in LV myocardium, expressed as the number of capillaries per field (a). Results are reported as mean ± SEM (*n* = 8/gr). Representative LV sections from sham-operated or AR rats showing isolectin B4-stained capillaries (brown-red) are illustrated.

Changes in the profile of LV gene expression in AR rats after 9 months of severe volume overload were evaluated using microarray analysis. Fold change level threshold between AR LV samples and sham controls was arbitrarily fixed to 1.5 times with a *p* value below 0.01 in order to consider a gene as regulated. Three hundred and ninety-four transcripts met these criteria (230 were upregulated and 164 were downregulated). As listed in Tables 4 and 5, gene ontology analysis showed that the most significantly upregulated gene categories were associated with extracellular space and matrix and the most downregulated were those associated with the mitochondria and metabolism (Tables S2 and S3). This general downregulation of genes associated with mitochondrial function was present for most of the enzymes implicated in the utilization of fatty acids as an energy substrate (Figures 1(a) and 1(b)). The microarray results were corroborated with quantitative RT-PCR determinations (Figures 1(b) and S1). Peroxisome proliferator activated receptor alpha (PPAR*α*) is the principal regulator of the expression of fatty acid *β*-oxidation FAO enzymes and transporters [28]. After 9 months of AR, gene expression of PPAR*α*, and its coactivator, PGC1*α* was strongly downregulated (Figure 1(c)). PPAR*α* binds to sequence-specific target elements as a heterodimer with the retinoid X receptor (RXR). In our microarray, we identified the RXR gamma isoform as the most expressed in the rat myocardium and the most downregulated in AR (not shown). We confirmed this using quantitative RT-PCR (Figure 1(c)). The other isoforms of RXR (alpha and beta) were also downregulated but less strongly.

### 3.4. Myocardial Capillaries Density

Long-term LV volume overload is associated with increased perivascular fibrosis as demonstrated before [9]. An additional factor that can influence oxygen and metabolic fuel availability and delivery to cardiomyocytes is capillaries density. Myocardial capillaries density was measured and the results can be found in Figure 1(d). Capillaries density was significantly lower in rats with aortic regurgitation after 9 months compared to the sham animals.

### 3.5. Mitochondrial DNA Content

Considering the important number of downregulated genes related to the mitochondria after 9 months of AR, we evaluated the amount of mitochondria in the LV of AR rats. To do so, the relative content of mitochondrial (mt) DNA was measured and compared to nucleus (n) DNA. The LV ratio of mtDNA to nDNA remained constant (sham: 3305 ± 130.5 units versus AR; 3276 ± 113.8) suggesting a stable proportion of mitochondria.

### 3.6. Fatty Acid Beta Oxidation (FAO) in Acute AR Rats

**Figure 2:**
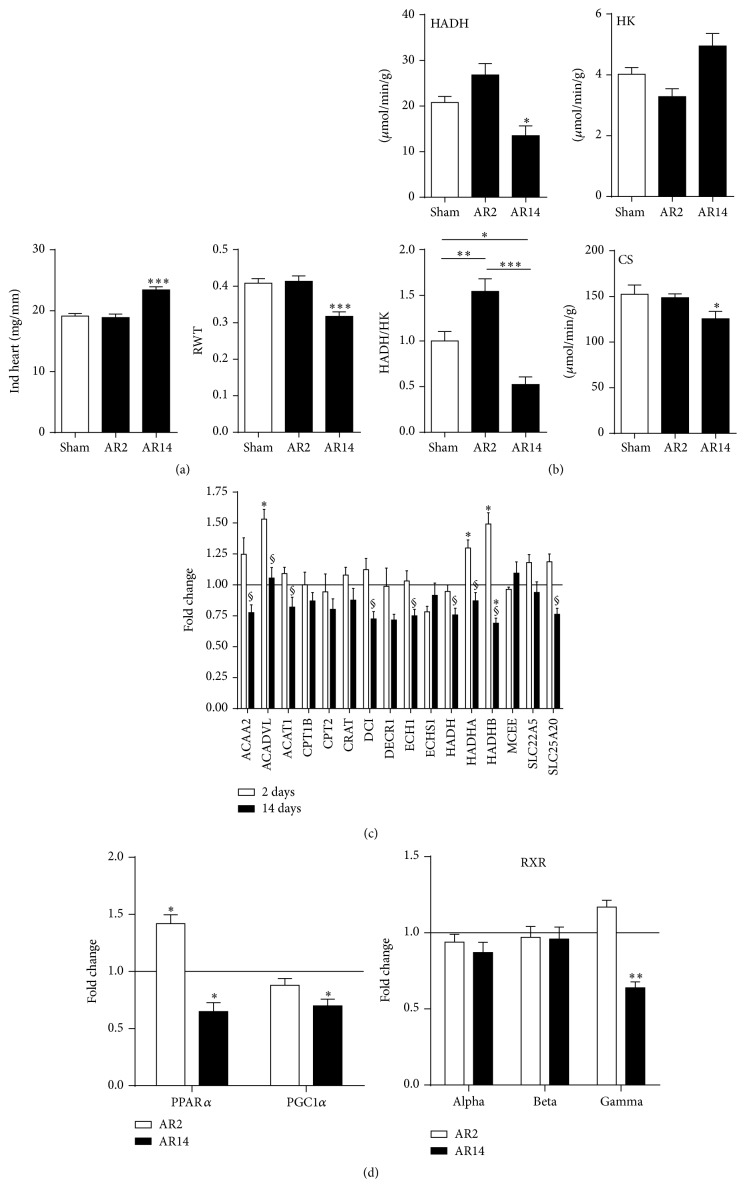
(a) Evolution of LV remodeling in experimental volume overload from severe aortic valve regurgitation in Wistar rats after 2 (AR2) and 14 days (AR14). Indexed heart weights were corrected for tibial length whereas relative wall thickness (RWT) was evaluated by echocardiography before sacrifice. Results are reported as the mean ± SEM (*n* = 6–8 per group). ^*∗∗∗*^ *p* < 0.001 between sham and AR groups. (b) LV myocardial activity levels of enzymes implicated in fatty acid *β*-oxidation, glucose metabolism, and mitochondrial energy production in 9-month AR rats relative to controls. HADH (hydroxyacyl-Coenzyme A dehydrogenase), HK (hexokinase), and citrate synthase (CS) enzymatic activities were measured in LV homogenates from at least 6 animals in each group as described in Section 2. Results are reported in *μ*moles/min/mg of tissue or as the ratio of HADH/HK activities arbitrarily fixed at 1 for sham group. ^*∗*^ *p* < 0.05, ^*∗∗*^ *p* < 0.01, and ^*∗∗∗*^ *p* < 0.001 between groups. (c) LV expression of the 16 genes studied in Figure 1(b) in AR rats at 2 and 14 days. Levels in sham animals were fixed to 1 (Line). ^*∗*^ *p* < 0.05 versus sham animals and ^§^ *p* < 0.05 between 2-day AR rats and 14-day animals. (d) PPAR*α*, PGC1*α*, and RXRs LV mRNA levels in 2- and 14-day AR rats. ^*∗*^ *p* < 0.05 and ^*∗∗*^ *p* < 0.01 compared to sham controls (Line at 1).

We then studied the expression of the same set of genes tested in the 9-month AR animals in the LV of rats with acute AR (2 and 14 days). As illustrated in Figure 2(a), heart hypertrophy had not already developed two days after AR whereas, after two weeks, indexed heart weight had increased by 22%. Eccentric LV remodeling as illustrated by the decrease of the relative wall thickness (as evaluated by echo) was also present. We measured the activity of a central enzyme in FAO, hydroxyacyl-Coenzyme A dehydrogenase (HADH), and the hexokinase (HK), the first step of glycolysis in myocardial tissue of AR rats 2 and 14 days after the surgery. As illustrated in Figure 2(b), a shift in the activity of these enzymes is apparent two weeks after AR, a period of very rapid and active development of LV hypertrophy and remodeling [17]. We did not observe this after 48 hours of AR although a trend for favoring increased FAO was present as demonstrated by an increased HADH/HK ratio. As for the expression of the FAO genes studied at 9 months (Figure 2(c)), the general downregulation begins to appear after two weeks of severe AR. Interestingly, several FAO genes (ACADVL, HADHA, and HAHDB) were upregulated 2 days after AR. LV gene expression for PPAR*α* and its activator PGC1*α* was downregulated at 14 days. On the other hand, at two days, PPAR*α* expression was significantly increased. The expression of RXR gamma followed a similar trend as illustrated in Figure 2(d).

### 3.7. Treatment with a PPAR*α* Agonist Can Reverse the Shift in Myocardial Substrate Preference Induced by LVH

**Figure 3:**
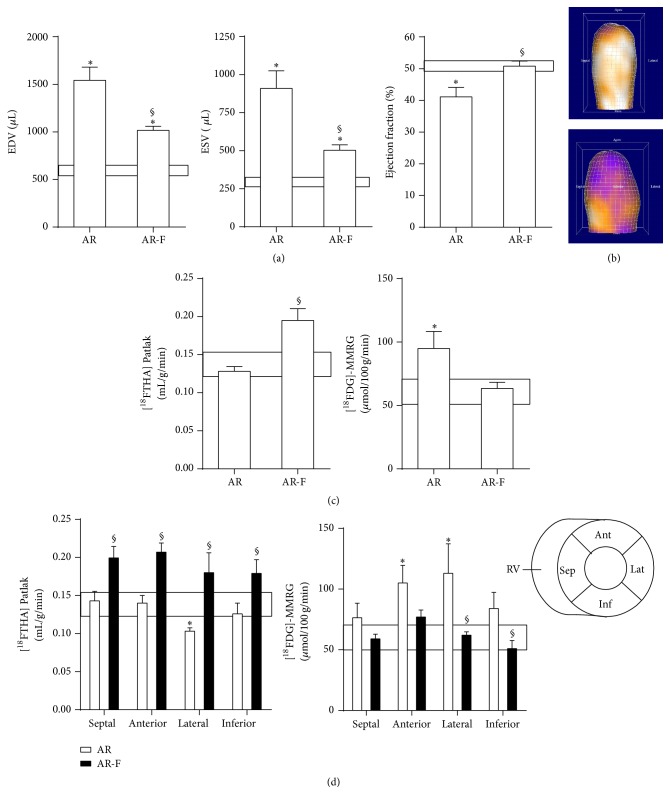
Impact of an 8-week fenofibrate treatment on LV remodeling, function, and energetics as evaluated by *μ*PET. (a) Left ventricular volumes and ejection fraction as evaluated by [^18^F]-FTHA *μ*PET. End-diastolic (EDV) and end-systolic (ESV) volumes were measured as described in Section 2. The ejection fraction is the ratio of SV (EDV-ESV) on EDV. Results are reported as the mean of data obtained from four animals/group ± SEM.: ^*∗*^ *p* < 0.05 between sham and AR groups. ^§^ *p* < 0.05 and untreated AR group. The box represents the mean ± SEM of sham animals. (b) A three-dimensional reconstruction of the LV of a sham (top) and an AR rat (bottom) 6 months after surgery. (d) The same analysis was then reproduced for each segment of the LV wall as schematized in the bottom right of this panel. ^*∗*^ *p* < 0.05 between sham and AR groups. Sept: septal wall, Ant: anterior wall, Lat: lateral wall, and Inf: inferior wall. ^*∗*^ *p* < 0.05 between sham and AR groups. ^§^ *p* < 0.05 and untreated AR group.

We showed recently that fenofibrate (a PPAR*α* agonist) can help reduce LV dilation in the AR rat model [15]. We studied* in vivo* the impact of fenofibrate on free fatty acid and glucose uptake by *μ*PET quantification as shown in Figure 3. This approach also allowed us to evaluate LV volumes and to measure the ejection fraction (EF). As illustrated in Figure 3(a), AR increased significantly both the end-diastolic (EDV) and end-systolic (ESV) LV volumes which resulted in a decreased EF compared to control sham-operated animals. Fenofibrate treatment reduced both EDV and ESV in AR animals and helped normalize the EF. A three-dimensional reconstruction of the LVs of a sham-operated and of an AR rat is illustrated in Figure 3(b).

The overall myocardial uptake of fatty acids in AR rats was similar to sham-operated animals. Fenofibrate treatment increased the myocardial avidity for [^18^F]-FTHA to supranormal levels (Figure 3(c)). On the other hand, glucose uptake by the LV of AR was significantly increased and this was reversed by fenofibrate. When the analysis was made on different LV regions (Figure 3(d)), fatty acid uptake was slightly decreased in the lateral wall (opposite to the septum) whereas glucose uptake was increased in both the lateral and the anterior walls. Fenofibrate treatment increased fatty acid uptake homogeneously in each LV wall. This was accompanied by a normalization of glucose uptake. Interestingly, at 8 weeks, fenofibrate upregulated the expression of a subset of the FAO genes studied (HADHB, ECI, ECH1, DECR1, ACAA2, and CPT2) in sham animals but not in AR rats (Figure S2).

## 4. Discussion

The factors influencing the development of eccentric LV hypertrophy from chronic VO and the evolution of the disease are poorly understood. In order to improve our knowledge of this condition, the need for animal models is important. As for many patients with significant AR, an important proportion of the AR rats can live more than a third of their normal lifespan with important heart dilation and without overt signs of HF. The study of chronic heart adaptations to hemodynamic overload in rodent models has received little attention in the past mainly for practical reasons (rapid evolution of some models toward HF, housing costs of larger protocol, etc.). Here, we present a gene profiling study of the left ventricles from an aging model of eccentric LVH after 9 months of severe AR.

We observed that many upregulated genes in the left ventricles of AR rats were linked to the extracellular matrix remodeling whereas those downregulated were often associated with myocardial metabolism. We had observed in a previous evaluation of the gene profile of left ventricles from rats after only 14 days of AR [8] that many genes associated with extracellular matrix remodeling were also upregulated very early in the disease process. This made sense considering that 14 days after AR corresponds to an early rapid LV remodeling phase in response to severe and acute LV volume overload [17, 29]. We had previously reported that the myocardial LV collagen tissue content in AR animals increased but only after 9 months [9]. An upregulation of genes related to the extracellular matrix is still present after 9 months suggesting a disruption in the balance between collagen synthesis and its degradation during the evolution of the disease. This probably takes place in the preceding months and leads to increase interstitial and perivascular fibrosis [9].

Eccentric LV hypertrophy is not normally associated with an accumulation of myocardial fibrosis at least during the early stages of VO. Myocardial collagen loss has even been observed [30, 31]. In the rat AR model, we did not observe such loss of collagen or downregulation of ECM genes in the early stages of the disease [29]. This can possibly be explained by an early pressure overload component often associated with AR at least before LV dilation has taken place. After 9 months, the presence of interstitial fibrosis is most likely linked to the loss and replacement of apoptotic myocytes by fibrotic tissue. This is accompanied with decreased myocardial relaxation as demonstrated by the decrease of *dP*/*dt*_min_. This could increase the occurrence of arrhythmias which we believe is the main cause of mortality in the AR rat model [9, 10, 13].

**Figure 4:**
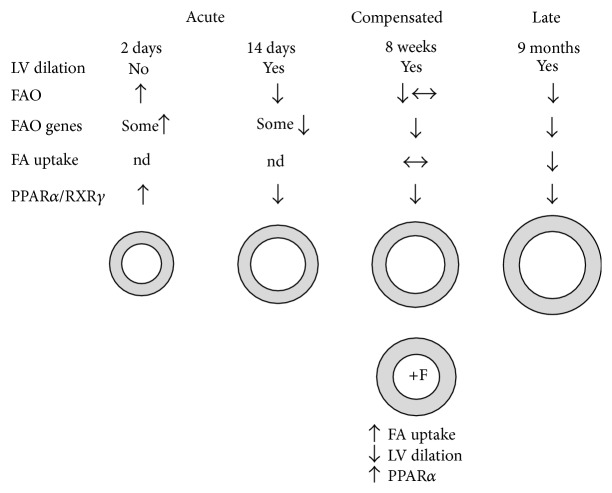
Summary of the observations related to myocardial FAO in the AR rat model made in this study. Soon after AR induction, FAO seems to be increased even before LV dilation has taken place. Then, FAO becomes downregulated as eccentric LVH develops. During the compensated phase of the disease, FAO is relatively stable although FAO genes are downregulated. Later in the disease, LV dilation is even more important. This is accompanied with a general decrease in fatty acids utilization by the heart. See Section 4 for additional information. F: fenofibrate. Nd: not determined.

We have summarized in Figure 4 most of the observations made in this study related to myocardial FAO in the AR rat mode. Soon after AR induction, FAO seems to be increased before LV dilation has taken place. Then, FAO becomes downregulated as eccentric LVH develops. During the compensated phase of the disease, glucose uptake is clearly above normal levels and seems to be the main way for the heart muscle to fuel its augmented energy needs. We had previously shown that, during this compensated phase at 8 weeks, myocardial oxidative metabolism was still unchanged compared to sham animals [26]. At 8 weeks, FA uptake is still normal or little decreased [26]. Then, late in the disease at 9 months, FAO is clearly downregulated both at the level of FA intake and HADH activity [14].

The present microarray analysis showed that an important number of downregulated genes was associated with the mitochondrial compartment confirming alterations in myocardial energetics in 9-month AR rats [14]. We had not clearly observed this in the microarray analysis we previously conducted from 14-day AR LVs [8]. Our results on acute AR rats confirm that FAO gene expression only begins to be downregulated two weeks after induction. It is intriguing that FAO activity seemed to be related to the state of LV dilation in acute AR rats. LV dilation can bring a state of ischemia if the increase in myocytes size is not accompanied with an activation of angiogenesis and the formation of new blood vessels. It is not the case in the AR myocardium as evidenced by our observation of a decreased capillary network. Glucose constitutes a less oxygen-consuming choice when LVH develops. Prior to this, at two days, increased FAO probably remains a more efficient option to fuel the heart with enough ATP. We had shown in the past that, during the first two days after AR induction, both LV inotropy and contractility were higher than normal to compensate for the sudden increase of blood to pump [17].

Heart FA uptake seemed to be maintained later in the disease as evidenced by the *μ*PET study. We only noticed a slight decrease in FA uptake after 8 weeks in the LV lateral wall. At this compensated phase of the disease, FAO enzyme activity is also only slightly reduced [12, 15]. In fact, we observed clearer differences here in the enzymatic activities related to FAO and glycolysis 2 weeks after AR induction than at 8 weeks in previous studies [12, 15, 26]. It is possible that, at two weeks, the intense LV remodeling necessitates an increased amount of energy whereas later, at 8 weeks, the LV has probably entered in a more stable and compensated phase of the disease.

A balance between FA uptake and utilization has to be stricken to avoid the accumulation of unwanted lipids in the cardiac muscle cell causing lipotoxicity. This has been observed in another model of eccentric LVH (mitral regurgitation in dogs) [32]. We reported in a previous study that the myocardial triglycerides content in 8-week AR rats was unchanged [12] and we did not observe positive staining for lipids using the oil red O method on LV section of 6-month AR rats (unpublished observation).

PPAR*α* and RXR*γ* gene expression mirrored the different observations we made on the state of myocardial FAO in AR rats at different times. PPARs dimerize with RXRs to bind to their sequence-specific target sequences. Our microarray and qRT-PCR data showed that RXR*γ* was the most highly expressed in the heart and that it was strongly downregulated in AR in parallel to PPAR*α* and PGC1*α* expression. Interestingly, PPAR*α* and RXR*γ* gene expression was upregulated early after AR induction again suggesting that FAO is first stimulated before glycolysis becomes more central in the energy production of the dilating heart.

The evaluation of energy substrates uptake* in vivo* in 8-week AR rats with or without treatment with fenofibrate showed that glucose uptake is clearly elevated before FA uptake decreased. This is an interesting observation indicating that, during the early phases of eccentric LVH, the heart relies on glucose to sustain its additional energy needs before FAO starts to decrease. Interestingly, FAO gene downregulation is also clearly present at 8 weeks whereas fatty acid uptake and oxidation are still relatively normal (Figure S2). This could be explained by a possible decrease in the protein turnover of the enzymes implicated in FAO. This hypothesis remains to be confirmed, however.

Fenofibrate treatment decreased LV dilation in our model. We had previously observed that LV weight was not reduced by fenofibrate treatment, but we observed that its remodeling tended to be more concentric with less chamber dilation and increased wall thickness [15]. Our present *μ*PET analysis did not contradict these previous observations and confirmed that fenofibrate can indeed limit LV dilation. If the fenofibrate effects on FA uptake can be associated with its PPAR*α* agonist activity, it is less clear if its effects on LV remodeling are completely mediated via PPAR*α* although some evidences in the literature point to this direction [33, 34]. We observed previously that fenofibrate restored PPAR*α* gene expression in AR rats [15]. PPAR*α* null mice develop more hypertrophy, production of more reactive oxygen species as well as an exaggerated production of extracellular matrix components [35, 36]. Treatment of PPAR*α* null mice with fenofibrate exacerbates LVH development in a pressure overload situation suggesting that the benefits observed here are mostly via PPAR*α* activation [37]. Fenofibrate can slow the development of LVH and protect the heart, namely, via its anti-inflammatory, antioxidant, and antifibrotic properties. Fenofibrate can also reduce the formation of endothelin-1, a prohypertrophic molecule [38–40]. We had showed previously that fenofibrate was able to reverse the decrease in HADH activity observed in the LV of 8-week AR rats [15]. Interestingly, 8-week AR rats treated with fenofibrate showed no upregulation of all the FAO genes studied whereas sham animals displayed an increase for several of them (Figure S2). It is possible that the response of FAO genes to fenofibrate becomes altered during the progression of the disease, however. One aspect we did not investigate here is the effects of fenofibrate towards the inflammatory component of hypertrophy. Many inflammation-related genes have been found to be upregulated late in the disease in rats with ACF [41]. Our microarray results also showed a number of genes associated with the inflammation (not shown). Fenofibrate has been shown to have beneficial impact by reducing myocardial inflammation in hypertrophy models which could limit its development [42, 43].

## 5. Study Limitations

The results of this study have to be viewed in the light of some limitations. Rodent heart metabolism may differ in some aspects from humans. This study relied mainly on the evaluation of gene expression levels and more thorough analysis at the level of protein content, activity, and localization are needed. The role of various signaling pathways in controlling the energy substrate preference shift involved in the development of eccentric LVH and metabolic alterations will need to be explored more in detail.

## 6. Conclusions

Our results clearly show that the myocardium with chronic VO sustains a significant metabolic stress and develops important energetics adaptations. These findings may improve our view of the dilated and hypertrophied hearts of patients with severe VO from valve disease. Clinicians currently follow those patients without any intervention for a good number of years, simply waiting for the LV to become too dilated, for the occurrence of symptoms or until systolic function begins to fall. Based on our findings, we suggest that those hearts develop severe metabolic abnormalities even when systolic function seems preserved and that intervention then can limit dilation and metabolic abnormalities. Focusing on myocardial metabolism by various interventions such as targeted drugs, specific diets, or exercise may help this metabolically stressed myocardium to improve its energy production and maybe prolong the preheart failure state significantly. Some of our previous studies support this view [9, 15, 26], but additional work will be needed to substantiate it.

## Supplementary Material

Additional materials and data. PCR primers used for the study of the concordance between the data obtained in the array and those from qRT-PCR for 11 up-regulated genes in 9-month AR rats. List of Gene Annotation System categories associated with up- and down-regulated genes identified from the array study. Finally, impact of an 8-week fenofibrate treatment on sham-operated and AR rats on the expression of FAO genes.

## Figures and Tables

**Table 1:** Primer assays used in Q-PCR analysis of gene expression.

mRNA	Symbol	Cat. number	Amplicon (bp)
Acetyl CoA acyltransferase 2	Acaa2	Rn.PT.58.5300756	111
Acyl CoA dehydrogenase, very long chain	Acadvl	Rn.PT.58.13279450	147
Acetyl CoA acetyltransferase 1	Acat1	Rn.PT.58.18447027	102
Carnitine O-acetyltransferase	Crat	Rn.PT.58.36282119	97
Carnitine palmitoyltransferase 1b, muscle	CPT1b	QT01084069	98
Carnitine palmitoyltransferase 2	CPT2	QT00186473	150
Cyclophilin a	Ppia	QT00177394	106
2,4-dienoyl CoA reductase 1	Decr1	Rn.PT.58.44352482	120
Enoyl-CoA hydratase 1	Ech1	Rn.PT.58.33832465	99
Enoyl-CoA delta isomerase 1	Eci1	Rn.PT.58.37662439	119
Hydroxyacyl-CoA dehydrogenase	Hadh	Rn.PT.58.17867024	135
Hydroxyacyl-CoA dehydrogenase alpha	Hadha	Rn.PT.58.46222281	138
Hydroxyacyl-CoA dehydrogenase beta	Hadhb	Rn.PT.58.7613498	130
Methylmalonyl CoA epimerase	Mcee	Rn.PT.58.10789169	101
Peroxisome proliferator activated receptor alpha	PPAR alpha	QT00176575	66
Peroxisome proliferator activated receptor gamma, coactivator 1 alpha	PGC1alpha	QT00189196	108
Retinoid X receptor alpha	Rxra	Rn.PT.58.33966638	103
Retinoid X receptor beta	Rxrb	Rn.PT.58.7033263	89
Retinoid X receptor gamma	Rxrg	Rn.PT.58.6519292	103
Solute carrier family 22, member 5	Slc22a5	Rn.PT.58.6675481	131
Solute carrier family 25, member 20	Slc25a20	Rn.PT.58.6247859	116

**Table 2:** Heart remodeling and hemodynamics at 9 months.

Parameters	Sham (*n* = 8)	AR (*n* = 8)	*p* value
Ind heart, mg/mm	21.3 ± 2.7	40.1 ± 1.6	<0.0001
SAP, mm Hg	120 ± 4.0	120 ± 3.3	0.84
DAP, mm Hg	90 ± 4.6	64 ± 2.0	<0.0001
PP, mm Hg	30 ± 2.1	56 ± 2.4	<0.0001
MAP, mm Hg	99 ± 4.3	83 ± 2.5	0.007
*dP*/*dt*_min⁡_, mm Hg/sec	−5994 ± 327	−3871 ± 143	<0.0001
*dP*/*dt*_max⁡_, mm Hg/sec	7483 ± 328	5657 ± 277	<0.0001
LVEDP, mm Hg	9.6 ± 1.6	14.4 ± 1.4	0.044

Measurements obtained under inhaled 1.5% isoflurane anesthesia in surviving animals. Ind heart: indexation was made using tibial length; SAP: systolic arterial pressure; DAP: diastolic arterial pressure; PP: pulse pressure (SAP-DAP); MAP: mean arterial pressure; *dP*/*dt*_min⁡_: minimal derivative of pressure/time; *dP*/*dt*_max⁡_: maximal derivative of pressure/time; LVEDP: left ventricular end-diastolic pressure. Values are mean ± SEM of the indicated number of animals with the exception of *dP*/*dt* and LVEDP values (*n* = 5).

**Table 3:** Echocardiographic data at 9 months.

	Sham (*n* = 8)	AR (*n* = 8)	*p* value
EDD, mm	9.2 ± 0.08	12.2 ± 0.24	<0.0001
ESD, mm	4.5 ± 0.07	7.6 ± 0.09	<0.0001
SW, mm	1.6 ± 0.03	1.8 ± 0.02	<0.0001
PW, mm	1.5 ± 0.42	1.8 ± 0.02	<0.0001
RWT	0.34 ± 0.004	0.29 ± 0.006	<0.0001
FS, %	51 ± 0.3	39 ± 1.3	<0.0001
SV, *µ*L	232 ± 4.8	372 ± 22.2	<0.0001

Measurements obtained under inhaled 1.5% isoflurane anesthesia after 9 months. EDD: end-diastolic diameter, ESD: end-systolic diameter, SW: septal wall, PW: posterior wall, RWT: relative wall thickness, FS: fractional shortening, and SV: stroke volume. Values are expressed as mean ± SEM of the indicated number of animals.

**Table 4:** Upregulated genes in the category “extracellular” from the GO cellular component in 9-month LVs from severe volume overload compared to age-matched sham-operated animals.

Target ID	Definition	Fold change	*p* value
NPPA	Natriuretic peptide precursor type A	6,775	0.00018
TGFB2	Transforming growth factor, beta 2	4,311	0.00005
CTGF	Connective tissue growth factor	4,059	0.00012
CHI3L1	Chitinase 3-like 1	3,775	0.00117
HAMP	Hepcidin antimicrobial peptide	3,397	0.00080
MGP	Matrix Gla protein	3,256	0.00011
LTBP2	Latent transforming growth factor beta binding protein 2	3,067	0.00073
TIMP1	Tissue inhibitor of metallopeptidase 1	2,954	0.00215
CTSS	Cathepsin S	2,912	0.00003
LOXL1	Lysyl oxidase-like 1	2,869	0.00047
PRSS23	Protease, serine, 23	2,788	0.00002
FSTL1	Follistatin-like 1	2,673	0.00033
GPX3	Glutathione peroxidase 3	2,672	0.00011
C1QB	Complement component 1, q subcomponent, beta polypeptide	2,613	0.00007
PTGIS	Prostaglandin I2 (prostacyclin) synthase	2,574	0.00005
SERPING1	Serine (or cysteine) peptidase inhibitor, clade G, member 1	2,520	0.00037
LGALS3	Lectin, galactose binding, soluble 3	2,419	0.00042
PMP22	Peripheral myelin protein 22	2,235	0.00025
IGFBP6	Insulin-like growth factor binding protein 6	2,231	0.00023
FN1	Fibronectin 1	2,230	0.00361
COL1A2	Procollagen, type I, alpha 2	2,212	0.00024
TGFA	Transforming growth factor alpha	2,177	0.00112
C1QA	Complement component 1, q subcomponent, alpha polypeptide	2,142	0.00013
ECM1	Extracellular matrix protein 1	2,098	0.00023
FBN1	Fibrillin 1	2,093	0.00056
MFAP4	Microfibrillar-associated protein 4	2,076	0.00009
FXYD6	FXYD domain-containing ion transport regulator 6	2,074	0.00115
PLOD2	Procollagen lysine, 2-oxoglutarate 5-dioxygenase 2	2,068	0.00021
WISP2	WNT1 inducible signaling pathway protein 2	2,060	0.00136
CTSK	Cathepsin K	2,051	0.00018
C1S	Complement component 1, s subcomponent	2,028	0.00210
APOE	Apolipoprotein E	2,027	0.00059
MXRA8	Matrix-remodelling associated 8	1,964	0.00027
NPPB	Natriuretic peptide precursor type B	1,924	0.00016
LUM	Lumican	1,902	0.00021
PCDH21	MT-protocadherin	1,861	0.00102
CD14	CD14 antigen	1,845	0.00003
TF	Transferrin	1,844	0.00089
C2	Complement component 2	1,807	0.00119
PPT1	Palmitoyl-protein thioesterase 1	1,753	0.00007
GDF15	Growth differentiation factor 15	1,705	0.00016
CX3CL1	Chemokine (C-X3-C motif) ligand 1	1,679	0.00108
AOC3	Amine oxidase, copper containing 3	1,666	0.00093
CCL7	Chemokine (C-C motif) ligand 7	1,665	0.00284
NBL1	Neuroblastoma, suppression of tumorigenicity 1	1,661	0.00048
GRN	Granulin	1,634	0.00010
SERPINF1	Serine (or cysteine) peptidase inhibitor, clade F, member 1	1,634	0.00038
CTSB	Cathepsin B	1,610	0.00005
FXYD5	FXYD domain-containing ion transport regulator 5	1,604	0.00167
TRH	Thyrotropin releasing hormone	1,588	0.00306
PRELP	Proline arginine-rich end leucine-rich repeat protein	1,580	0.00127
STC1	Stanniocalcin 1	1,550	0.00049
COL5A1	Procollagen, type V, alpha 1	1,536	0.00065
CD48	CD48 antigen	1,533	0.00047
PON3	Paraoxonase 3	1,522	0.00508
ITGB1	Integrin beta 1	1,519	0.00002
RARRES2	Retinoic acid receptor responder	1,509	0.00004

FC: fold change versus sham controls.

**Table 5:** Downregulated genes in the category “mitochondrion” from the GO cellular component in 9-month LVs from severe volume overload compared to age-matched sham-operated animals.

Target ID	Definition	Fold change	*p* value
CYP11A1	Cytochrome P450, family 11, subfamily a, polypeptide 1	0,482	0.00057
ECH1	Enoyl-Coenzyme A hydratase 1, peroxisomal	0,497	0.00039
HADHA	Hydroxyacyl-Coenzyme A dehydrogenase/3-ketoacyl-Coenzyme A thiolase/enoyl-Coenzyme A hydratase (trifunctional protein), alpha subunit	0,517	0.00030
DECR1	2,4-Dienoyl CoA reductase 1, mitochondrial	0,519	0.00343
ACAA2	Acetyl-Coenzyme A acyltransferase 2 (mitochondrial 3-oxoacyl-Coenzyme A thiolase)	0,523	0.00536
LDHD	Lactate dehydrogenase D	0,540	0.00088
DCI	Dodecenoyl-coenzyme A delta isomerase	0,541	0.00037
ACAT1	Acetyl-coenzyme A acetyltransferase 1	0,541	0.00023
PKM2	Pyruvate kinase, muscle	0,547	0.00020
MLYCD	Malonyl-CoA decarboxylase	0,554	0.00111
BCAT2	Branched chain aminotransferase 2, mitochondrial	0,557	0.00184
GSTK1	Glutathione S-transferase kappa 1	0,572	0.00035
FAHD1	Fumarylacetoacetate hydrolase domain containing 1	0,581	0.00008
DHRS4	Dehydrogenase/reductase (SDR family) member 4	0,585	0.00035
HSD17B8	Hydroxysteroid (17-beta) dehydrogenase 8	0,585	0.00018
ACSL1	Acyl-CoA synthetase long-chain family member 1	0,589	0.00023
BCKDHA	Branched chain ketoacid dehydrogenase E1, alpha polypeptide	0,593	0.00109
ACADVL	Acyl-Coenzyme A dehydrogenase, very long chain	0,597	0.00089
SLC25A20	Solute carrier family 25 (mitochondrial carnitine/acylcarnitine translocase), member 20	0,598	0.00021
ACO2	Aconitase 2, mitochondrial	0,601	0.00173
LOC56764	DNAJ-like protein	0,607	0.00045
PECR	Peroxisomal trans-2-enoyl-CoA reductase	0,610	0.00033
ECHS1	Enoyl-Coenzyme A hydratase, short chain, 1, mitochondrial	0,616	0.00065
IVD	Isovaleryl coenzyme A dehydrogenase	0,618	0.00098
PDK2	Pyruvate dehydrogenase kinase, isoenzyme 2	0,622	0.00071
MGST1	Microsomal glutathione S-transferase 1	0,623	0.00001
CRAT	Carnitine acetyltransferase	0,630	0.00125
ACADS	Acetyl-Coenzyme A dehydrogenase, short chain	0,635	0.00507
SUCLG1	Succinate-CoA ligase, GDP-forming, alpha subunit	0,637	0.00048
NDUFS7	NADH dehydrogenase (ubiquinone) Fe-S protein 7	0,638	0.00280
NDUFA10	NADH dehydrogenase (ubiquinone) 1 alpha subcomplex 10	0,654	0.00101
IDH3G	Isocitrate dehydrogenase 3 (NAD), gamma	0,655	0.00041
ATAD3A	ATPase family, AAA domain containing 3A	0,658	0.00060
RGD735029	SEL1 domain containing protein	0,659	0.00097
RGD1303003	Homolog of zebrafish ES1	0,661	0.00011
RGD1303272	Similar to RIKEN cDNA 2010311D03	0,662	0.00218
HADHB	Hydroxyacyl-Coenzyme A dehydrogenase/3-ketoacyl-Coenzyme A thiolase/enoyl-Coenzyme A hydratase (trifunctional protein), beta subunit	0,662	0.00078
CS	Citrate synthase	0,663	0.00559
GRPEL1	GrpE-like 1, mitochondrial	0,666	0.00101
PDP2	Pyruvate dehydrogenase phosphatase isoenzyme 2	0,668	0.00489
HSD17B10	Hydroxysteroid (17-beta) dehydrogenase 10	0,670	0.00185

FC: fold change versus sham controls.
