# Supplementary material for: Transcriptional Changes Associated with Long-Term Left Ventricle Volume Overload in Rats: Impact on Enzymes Related to Myocardial Energy Metabolism
Source: Biomed Res Int. 2015 Oct 25;2015:949624. doi: 10.1155/2015/949624 (PMC4637065; doi:10.1155/2015/949624)
Supplement: Supplementary file 1 — Additional materials and data. PCR primers used for the study of the concordance between the data obtained in the array and those from qRT-PCR for 11 up-regulated genes in 9-month AR rats. List of Gene Annotation System categories associated with up- and down-regulated genes identified from the array study. Finally, impact of an 8-week fenofibrate treatment on sham-operated and AR rats on the expression of FAO genes. [file 949624.f1.pdf]

Data supplement

Transcriptional changes associated with long-term left ventricle volume overload in rats: impact on enzymes related to myocardial energy metabolism.

Elise Roussel, Marie-Claude Drolet, Elisabeth Walsh-Wilkinson, Wahiba Dhahri, Dominic Lachance, Suzanne Gascon*, Otman Sarrhini*, Jacques A. Rousseau*, Roger Lecomte*, Jacques Couet and Marie Arsenault.

Groupe de recherche sur les valvulopathies, Centre de Recherche, Institut universitaire de cardiologie et de pneumologie de Québec, Université Laval, Quebec City, Canada.
*: Centre d'imagerie moléculaire de Sherbrooke, Centre de recherché Étienne-LeBel, Centre Hospitalier Universitaire de Sherbrooke, Université de Sherbrooke, Sherbrooke, Canada.

Corresponding authors: Jacques Couet PhD and Marie Arsenault MD
Groupe de Recherche en Valvulopathies, Centre de Recherche,
Institut universitaire de cardiologie et de pneumologie de Québec
2725, Chemin Sainte-Foy, Québec, (Québec), Canada, G1V 4G5
Phone: 1-418-656-4760; Fax: 1-418-656-4509
Email: jacques.couet@med.ulaval.ca or marie.arsenault@criucpq.ulaval.ca

Table S1. Primer Assays used in Q-PCR analysis of gene expression (Figure S1).

mRNA	Symbol	Cat. No.	Amplicon (bp)
Connective tissue growth factor	Ctgf	QT00182021	102
Cathepsin K	Ctsk	QT00375599	80
Fibronectin 1	Fn1	QT00179333	92
Follistatin-like 1	Fstl1	QT00195762	131
Hepcidin antimicrobial peptide	Hamp	QT00372876	120
Integrin beta 1	Itgb1	QT00187656	117
Natriuretic peptide precursor type A	Nppa	QT00366170	94
Natriuretic peptide precursor type B	Nppb	QT00183225	89
Peripheral myelin protein 22	Pmp22	QT00175938	89
Transforming growth factor beta 2	Tgfb2	QT00187320	139
Tissue inhibitor of metallopeptidase 1	Timp1	QT00185304	113

Table S2. Significant up-regulated gene categories based on gene ontology annotation system.

System	Gene Category	No. of genes	EASE score[a]
GO Biological Process	regulation of cell cycle	16	4.29E-04
GO Biological Process	cell growth	9	5.59E-04
GO Biological Process	transition metal ion homeostasis	5	6.27E-04
GO Biological Process	di-, tri-valent inorganic cation homeostasis	7	7.50E-04
GO Biological Process	response to biotic stimulus	23	8.05E-04
GO Molecular Function	calcium ion binding	25	6.90E-06
GO Molecular Function	metal ion binding	36	8.01E-05
GO Molecular Function	cell adhesion molecule activity	15	2.11E-04
GO Molecular Function	calcium-dependent phospholipid binding	5	6.74E-04
GO Cellular Component	extracellular	70	2.70E-11
GO Cellular Component	extracellular matrix	22	6.04E-08
GO Cellular Component	extracellular space	42	3.80E-06

a: Ease Probability score. E: exponent, power of 10.

Table S3. Significant down-regulated gene categories based on gene ontology annotation system.

System	Gene Category	No. of genes	EASE score[a]
GO Biological Process	organic acid metabolism	18	2.49E-07
GO Biological Process	carboxylic acid metabolism	18	2.49E-07
GO Biological Process	fatty acid metabolism	11	4.64E-06
GO Biological Process	energy derivation by oxidation of organic compounds	10	2.75E-05
GO Biological Process	energy pathways	11	2.89E-05
GO Molecular Function	oxidoreductase activity	26	1E-10
GO Molecular Function	transferase activity	26	3.06E-04
GO Cellular Component	mitochondrion	54	4.8E-24

a: Ease Probability score. E: exponent, power of 10.

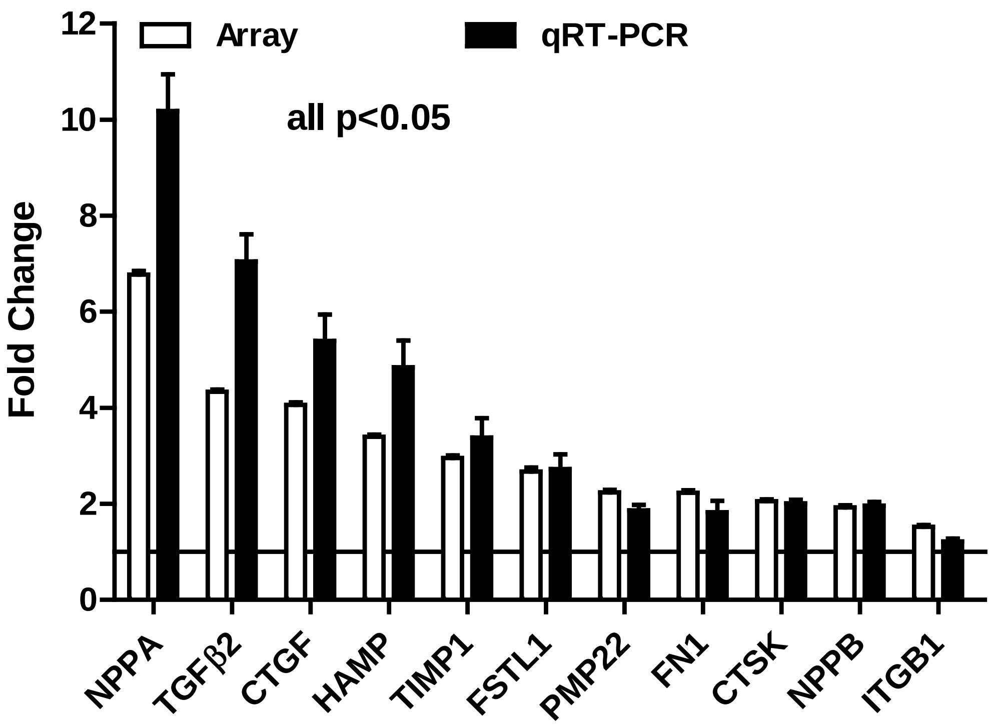

Figure S1. Comparison between fold change results obtained from the microarray and by quantitative RT-PCR for a subset of 11 up-regulated genes as determined in the array study. Results are reported in as mean ± SEM (n = 5 per group for array and n=6 for qRT-PCR). Levels in sham animals were fixed to 1.

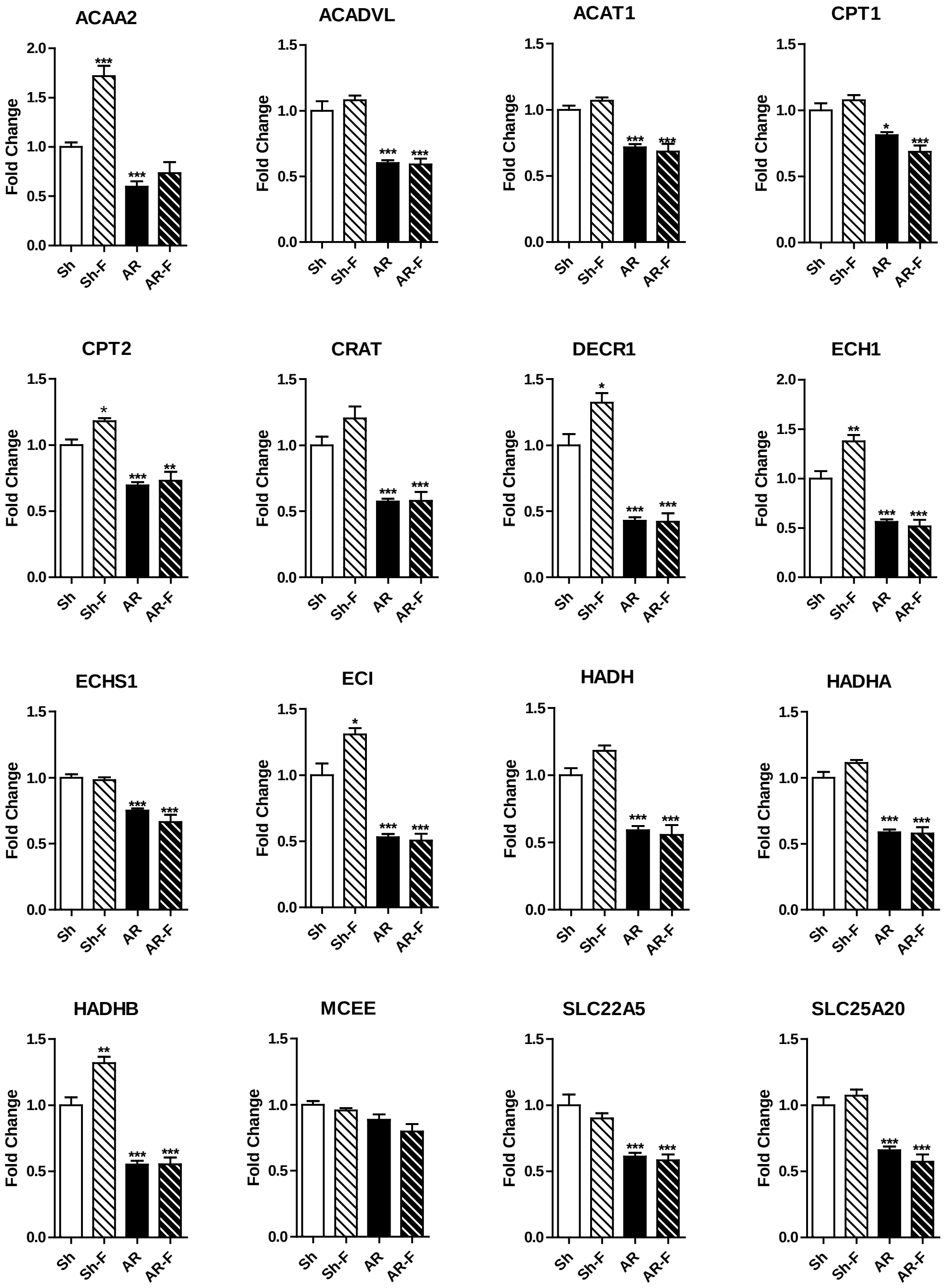

Figure S2. Impact of an 8-week fenofibrate (F) treatment on LV expression of 16 FAO genes in Sham (Sh) and AR rats. Levels in sham animals were fixed to 1 (Line). *: p<0.05, **: p<0.01 and ***: p<0.001 vs. untreated sham animals.
